# Clinical practice of language fMRI in epilepsy centers: a European survey and conclusions by the ESNR Epilepsy Working Group

**DOI:** 10.1007/s00234-020-02397-w

**Published:** 2020-03-13

**Authors:** N. Bargalló, I. Cano-López, C. Rosazza, M. W. Vernooij, M. Smits, P. Vitali, J. Alvarez-Linera, H. Urbach, L. Mancini, A. Ramos, T. Yousry

**Affiliations:** 1 Magnetic Resonance Image Core Facility, IDIBAPS and Center of Diagnostic Image (CDIC), Hospital Clinic, Barcelona, Spain; 2 Valencian International University, Valencia, Spain; 3 Neuroradiology Unit, Fondazione IRCCS Istituto Neurologico “Carlo Besta”, Milan, Italy; 4 Department of Radiology & Nuclear Medicine, Erasmus MC University Medical Center, Rotterdam, The Netherlands; 5 Neuroradiology and Brain MRI 3T Mondino Research Center, IRCCS Mondino Foundation, Pavia, Italy; 6 Neuroradiology Department, Hospital Ruber Internacional, Madrid, Spain; 7 Department of Neuroradiology, Freiburg University Medical Center, Freiburg (i.Br.), Germany; 8 Lysholm Department of Neuro-radiology, National Hospital for Neurology and Neurosurgery, University College London Hospitals NHS Trust, London, UK; 9 Departments Radiology (A.H., A.R.), Hospital Universitario 12 de Octubre, Madrid, Spain

**Keywords:** fMRI, Language, Epilepsy, Survey, Europe

## Abstract

**Purpose:**

To assess current clinical practices throughout Europe with respect to acquisition, implementation, evaluation, and interpretation of language functional MRI (fMRI) in epilepsy patients.

**Methods:**

An online survey was emailed to all European Society of Neuroradiology members (*n* = 1662), known associates (*n* = 6400), and 64 members of European Epilepsy network. The questionnaire featured 40 individual items on demographic data, clinical practice and indications, fMRI paradigms, radiological workflow, data post-processing protocol, and reporting.

**Results:**

A total of 49 non-duplicate entries from European centers were received from 20 countries. Of these, 73.5% were board-certified neuroradiologists and 69.4% had an in-house epilepsy surgery program. Seventy-one percent of centers performed fewer than five scans per month for epilepsy. The most frequently used paradigms were phonemic verbal fluency (47.7%) and auditory comprehension (55.6%), but variants of 13 paradigms were described. Most centers assessed the fMRI task performance (75.5%), ensured cognitive-task adjustment (77.6%), trained the patient before scanning (85.7%), and assessed handedness (77.6%), but only 28.6% had special paradigms for patients with cognitive impairments. fMRI was post-processed mainly by neuroradiologists (42.1%), using open-source software (55.0%). Reporting was done primarily by neuroradiologists (74.2%). Interpretation was done mainly by visual inspection (65.3%). Most specialists (81.6%) were able to determine the hemisphere dominance for language in more than 75% of exams, attributing failure to the patient not performing the task correctly.

**Conclusion:**

This survey shows that language fMRI is firmly embedded in the preoperative management of epilepsy patients. The wide variety of paradigms and the use of non-CE-marked software underline the need for establishing reference standards.

**Electronic supplementary material:**

The online version of this article (10.1007/s00234-020-02397-w) contains supplementary material, which is available to authorized users.

## Introduction

Epilepsy is one of the most common neurological diseases globally, affecting more than 70 million people worldwide [1]. While most patients with epilepsy achieve seizure control with antiepileptic drugs (AEDs), approximately 30% of patients have drug-resistant epilepsy [2, 3]. Temporal lobe epilepsy (TLE) is the most common type of drug-resistant epilepsy in adults [4] and surgical resection of the epileptogenic focus is often the appropriate treatment to achieve seizure control [5, 6].

Presurgical assessment of candidates for TLE surgery includes determining the language dominant hemisphere to estimate the postoperative risk of language and memory loss. The Wada test has been the gold standard [7], but is being increasingly replaced by functional MRI (fMRI). This non-invasive technique is now widely used in many epilepsy centers to assess language lateralization [8]. A recent survey from the European Union’s E-PILEPSY project reported that 82% of European epilepsy centers use language fMRI, primarily when the suspected epileptogenic zone is close to eloquent cortex [9].

The use of fMRI to determine language lateralization is a challenge in epilepsy patients because of (1) the relatively high prevalence of atypical (bilateral or right-hemispheric) language representation of up to 33% [10], (2) frequently co-occurring cognitive impairment [11], which can limit the patient’s performance when using the most prevalent language paradigms [12], and (3) the high variability of the paradigms used [13]. Despite fMRI’s clinical utility and widespread use, only scarcely recommendations have been made on the use of this technique [9, 14, 15].

Two studies surveyed the utility, implementation, and efficacy of presurgical language fMRI in epilepsy surgery centers [16, 17]. As the largest contribution came from US centers (almost two-thirds in Benjamin et al. [17] and 44% in Benjamin et al. [16], the applicability of the findings to European centers is uncertain.

An epilepsy working group was established by the Diagnostic Committee of the European Society of Neuroradiology (ESNR) with the aim of assessing the current clinical practice of using fMRI to determine language lateralization in adults with drug-resistant epilepsy. A European-wide survey was distributed by the Epilepsy Working Group among ESNR members and affiliates, querying the current practices throughout Europe with respect to implementation, evaluation, and interpretation of fMRI exams for language lateralization in epilepsy patients. The results of this survey, as well as conclusions by the Working Group, are reported in this manuscript.

## Methods

An online survey was designed using Google forms open-access toolbox (Google.com, Mountainview, CA, USA). Questions were assembled by the members of the ESNR Working Group (Lead NB). The questionnaire featured 40 individual items, divided into multiple-choice, single best choice, and free text answers (supplementary material). Information was gathered on demographic data, clinical practice and indications, fMRI paradigms, radiological workflow, data post-processing protocol, and reporting.

Survey invitations were emailed to ESNR members (*n* = 1662), known associates (*n* = 6400), and 64 members of the European Epilepsy network (EpiCARE). Only the participants with experience in the clinical use of language fMRI in epilepsy patients were invited to fill the survey. The survey was launched in November 2017 and concluded in April 2018. To avoid duplicate bias, participants were instructed to supply institution details. Fifty-five surveys were received. Three responders reported that they do not perform clinical language fMRI and two responders did not fill out the questionnaire; these five responses were removed from the analysis. Additionally, one responder sent the survey twice; the duplicate was removed. The results of the survey were presented by the members of the Epilepsy Working Group at the 2018 annual meeting of the ESNR in Rotterdam, The Netherlands.

Descriptive statistics were performed using IBM SPSS 22.

## Results

### Demographic data, clinical practice, and indications

Considering that multiple responses were possible for some questions, we provide the figures (*n*/total responses given) and percentages of each question.

**Fig. 1:**
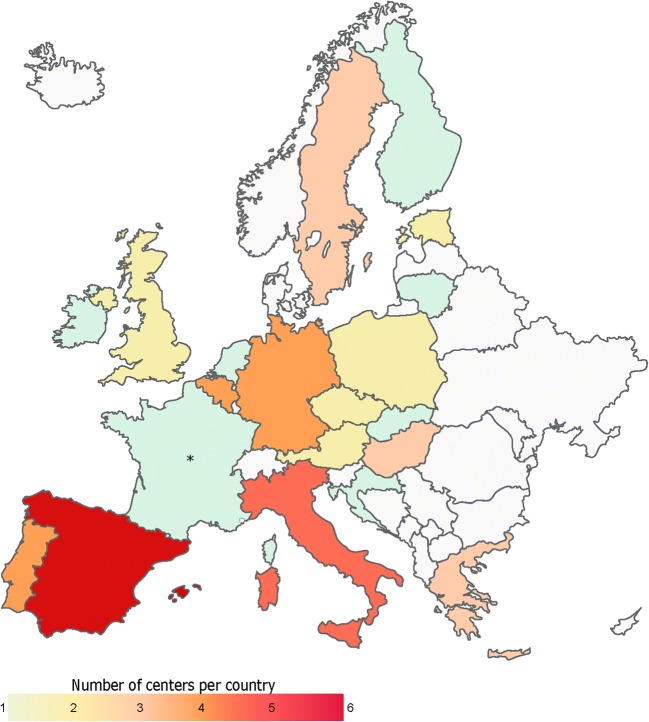
Institutional responses (number) per country. Countries with no responses are shaded gray. An asterisk indicates responding center located in Réunion (an overseas department and region of France, member of the European Union, and an island in the Indian Ocean, east of Madagascar and southwest of Mauritius)

Completed questionnaires were received from 49 unique (non-duplicate) European centers, out of a total of 20 countries. Figure 1 shows a map of the distribution of responses per country. Countries with the highest number of participating institutions were Spain (6), Italy (5), Belgium (4), Germany (4), and Portugal (4).

Out of the 49 respondents, 36 (73.5%) were neuroradiologists, 5 (10.2%) general radiologists, 2 (4.1%) neuroradiologists in training, 2 (4.1%) neurologists, 1 (2.0%) neuropsychologist, 1 (2.0%) neurobiologist, 1 (2.0%) biomedical engineer, and 1 (2.0%) did not specify their specialty. Thirty-six (73.5%) worked in academic hospitals and 13 (26.5%) in non-academic centers (10 [20.4%] general hospitals, 2 [4.1%] diagnostic neuroradiology centers, and 1 [2.0%] private hospital). Furthermore, 34 (69.4%) had an in-house epilepsy surgery service.

**Fig. 2:**
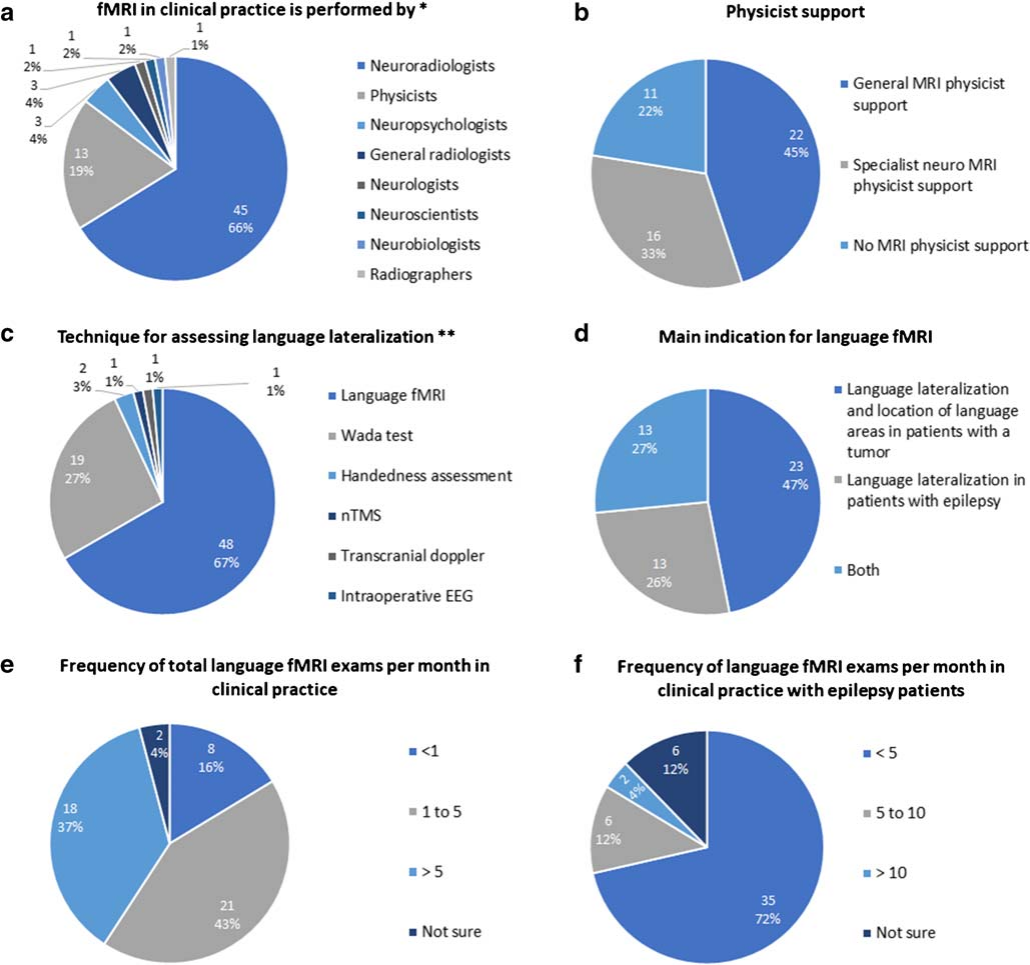
Clinical practice and indications for language fMRI. **a** Specialists who perform fMRI. **b** Physicist support. **c** Techniques used for assessing language lateralization. **d** Main indication for language fMRI. **e** Frequency of total language fMRI exams per month. **f** Frequency of language fMRI exams per month with epilepsy patients. EEG electroencephalogram, nTMS navigated transcranial magnetic stimulation, *total responses = 68, **total responses = 67

Clinical fMRI was mostly performed by neuroradiologists (45/68, 66.2%) and physicists (13/68, 19.1%) (Fig. 2a; note that multiple answers were possible to this question). Physicist support was available in 38/49 (77.6%) centers (Fig. 2b).

Language fMRI was the most widely used technique for assessing hemispheric dominance for language (48/67, 71.6%) followed by the Wada test (14/67, 20.9%) (Fig. 2c; note that multiple answers were possible for this question).

The main indication for clinical language fMRI, however, was determining language lateralization and location of language areas in patients with tumors (23/49, 46.9%) (Fig. 2d). Out of 49 centers, 21 performed 1–5 (21/49, 42.9%) examinations per month irrespective of the indication (Fig. 2e) and 35 centers (71.4%) performed 1–5 examinations per month for epilepsy patients (Fig. 2f). Out of the 19 centers that used the Wada test, thirteen (68.4%) performed fewer than five per year.

### Language fMRI paradigms and radiological workflow

Out of 49 centers, language lateralization was assessed using one paradigm in 6 (12.2%) centers and more than one in 43 (87.7%) centers (two paradigms in 23 [46.9%], three in 12 [24.5%] and four or more in 8 [16.3%] centers). Regarding language areas, 24 (49.0%) centers used separate paradigms to identify inferior frontal (includes Broca’s area) and superior temporal language areas (includes Wernicke’s area), 25 (51.0%) centers used one paradigm to identify both regions, and one center (2.0%) used additional paradigms to identify posterior temporal areas (picture and auditory naming paradigms). The most frequently used paradigm for inferior frontal area activation was phonemic verbal fluency (21/44 responses, 47.7%) whereas auditory comprehension (15/27 responses, 55.6%) for superior temporal area activation was used (Table 1). Variations of 13 standard language fMRI paradigms were reported, with the main differences being in their baseline task (Table 2).

**Table 1:** Type of language fMRI paradigms used (multiple answers were possible)

Type of paradigm	Percentage	*N*/total
Inferior frontal area activation (includes Broca’s area)		
Phonemic verbal fluency	47.7	21/44
Semantic verbal fluency (categories)	25.0	11/44
Verb generation	13.6	6/44
Picture naming	9.1	4/44
Verb to noun generation	2.3	1/44
Antonym generation	2.3	1/44
Superior temporal area activation (includes Wernicke’s area)		
Auditory comprehension (listening stories)	55.6	15/27
Reading	14.8	4/27
Semantic verbal fluency (categories)	7.4	2/27
Verb generation	3.7	1/27
Auditory naming	3.7	1/27
Auditory comprehension (listening sentences)	3.7	1/27
Verb to noun generation	3.7	1/27
Picture naming	3.7	1/27
Sentence evaluation test (right or wrong)	3.7	1/27
Simultaneous inferior frontal area and superior temporal area activation (includes Broca’s and Wernicke’s areas)		
Phonemic verbal fluency	20.5	9/44
Semantic verbal fluency (categories)	13.6	6/44
Verb to noun generation	11.4	5/44
Auditory comprehension (listening stories)	9.1	4/44
Auditory naming	6.8	3/44
Word pairing	6.8	3/44
Word decision task	6.8	3/44
Verb generation	4.5	2/44
Synonym decision task	4.5	2/44
Sentence completion	4.5	2/44
Picture naming	2.3	1/44
Proverbs	2.3	1/44
Go/no-go task	2.3	1/44
Repetition	2.3	1/44
Auditory comprehension (listening sentences)	2.3	1/44

**Table 2:** Variants of the most commonly used paradigms reported by centers

Paradigm	Activated areas	Activation task	*N*/total (%)	Control task	*N*/total (%)
Phonemic verbal fluency	Inferior frontal and superior temporal areas (includes Broca’s and Wernicke’s areas)	A letter is auditory or visually presented and patient is asked to think of words which start by the letter (e.g., F, A, S, or C, T, R), either silently or vocally	18/49 (36.7%)	Eyes closed rest	8/18 (44.4%)
				Eyes open rest, black screen displayed	1/18 (5.6%)
				Eyes open rest, crosshair fixation	4/18 (22.2%)
				Eyes open rest, dot	1/18 (5.6%)
				Imagine your favorite place	1/18 (5.6%)
				To think to a nonsense word	2/18 (11.1%)
				Finger tapping	1/18 (5.6%)
Semantic verbal fluency	Inferior frontal and superior temporal areas (includes Broca’s and Wernicke’s areas)	A category is auditory or visually presented and patient is asked to think of words belonging to that category (e.g., animals, foods, countries), either silently or vocally	7/49 (14.3%)	Eyes open rest, crosshair fixation	1/7 (14.3%)
				Eyes open rest, symbols	1/7 (14.3%)
				Imagine your favorite place	1/7 (14.3%)
				Counting	2/7 (28.6%)
				Finger tapping	2/7 (28.6%)
Verb generation	Inferior frontal and superior temporal areas (includes Broca’s and Wernicke’s areas)	A noun is auditory or visually presented and patient is asked to think of verbs associated with it, either silently or vocally	13/49 (26.5%)	Eyes closed rest	2/13 (15.4%)
				Eyes open rest, crosshair fixation	1/13 (7.7%)
				Eyes open rest, symbols	2/13 (15.4%)
				A nonsense word	2/13 (15.4%)
				Silent reading/repetition of adjective	3/13 (23.1%)
				Silent repetition of noun	1/13 (7.7%)
				Counting	1/13 (7.7%)
				Finger tapping	1/13 (7.7%)
Verb to noun generation	Inferior frontal and superior temporal areas (includes Broca’s and Wernicke’s areas)	A verb is auditory or visually presented and patient is asked to think of nouns associated with it, either silently or vocally	3/49 (6.1%)	Eyes closed rest	2/3 (66.7%)
				Passive viewing of a string of nonsense characters	1/3 (33.3%)
Picture naming	Inferior frontal and superior temporal areas (includes Broca’s and Wernicke’s areas)	A picture is presented, and patient is asked to name the object	5/49 (10.2%)	Eyes open rest, crosshair fixation	2/5 (40.0%)
				Eyes open rest, black screen displayed	1/5 (20.0%)
				Scrambled images	1/5 (20.0%)
				Decide what is the direction of the arrow	1/5 (20.0%)
Auditory naming	Inferior frontal and superior temporal areas (includes Broca’s and Wernicke’s areas)	Description of an object is auditory presented, and patient is asked to name the object	2/49 (4.0%)	Counting	1/2 (50.0%)
				Think: “I have to stay still”	1/2 (50.0%)
Auditory comprehension, passive	Inferior frontal and superior temporal areas (includes Broca’s and Wernicke’s areas)	Auditory stimuli (e.g., words, sentences, stories) are presented and no response is required	10/49 (20.4%)	Listening to white noise	3/10 (30.0%)
				Listening to music	1/10 (10.0%)
				Reverse speech	6/10 (60.0%)
Auditory comprehension, active	Inferior frontal and superior temporal areas (includes Broca’s and Wernicke’s areas)	Auditory stimuli (e.g., words, sentences, stories) are presented and a response is required	1/49 (2.0%)	Active listening to the same story played backward (“nonsense”)	1/1 (100.0%)
Synonym decision task	Inferior frontal and superior temporal areas (includes Broca’s and Wernicke’s areas)	Two words are auditory or visually presented and patient is asked to judge whether words are synonyms or not	4/49 (8.1%)	Decide whether two nonwords are synonyms or not	4/4 (100.0%)
Semantic decision task: category judgment	Inferior frontal and superior temporal areas (includes Broca’s and Wernicke’s areas)	Two words are auditory or visually presented and patient is asked to judge whether words belong to the same semantic category	3/49 (6.1%)	Judge if two geometric figures are similar or not	1/3 (33.3%)
				Judge if two letters are similar or not	1/3 (33.3%)
				Finger tapping	1/3 (33.3%)
Semantic decision task: sentence judgment	Superior temporal area (includes Wernicke’s area)	A complete sentence is presented, and patient is asked to judge whether the sentence makes sense or not	3/49 (6.1%)	Judge if two geometric figures are similar or not	1/3 (33.3%)
				Tone discrimination	1/3 (33.3%)
				Finger tapping	1/3 (33.3%)
Reading	Superior temporal area (includes Wernicke’s area)	Text is visually presented, and patient is asked to imagine vocalizing text silently	6/49 (1.2%)	Eyes closed rest	2/6 (33.3%)
				Eyes open rest, checkerboard displayed	1/6 (16.7%)
				Eyes open rest, crosshair fixation	1/6 (16.7%)
				Same text played backward	2/6 (33.3%)
Sentence completion	Inferior frontal and superior temporal areas (includes Broca’s and Wernicke’s areas)	A sentence is presented, and patient is asked to generate the final word	2/49 (4.0%)	Eyes closed rest	1/2 (50.0%)
				Listening to the sentence “I have to stay” and think “still”	1/2 (50.0%)

The paradigms’ stimuli were most frequently presented visually (38/68, 55.9%), followed by auditory (29/68, 42.6%) and tactile presentation (1/68, 1.5%) (note that multiple answers were possible to this question). Word generation paradigms were performed in 47/49 (95.9%) centers, most often in silence (without pronouncing the words: 40/47, 85.1%), but sometimes overtly (speaking normally: 4/47, 8.5%; or whispering: 3/47, 6.4%).

Centers used a wide variety of control tasks in each paradigm. The most common control tasks used were eyes closed or eyes open looking at a crosshair (each was used in 5 of the 13 described paradigms). Other control tasks were finger tapping (used in 4 paradigms) and counting and reverse speech listening (used in 3 paradigms) (Table 2).

**Fig. 3:**
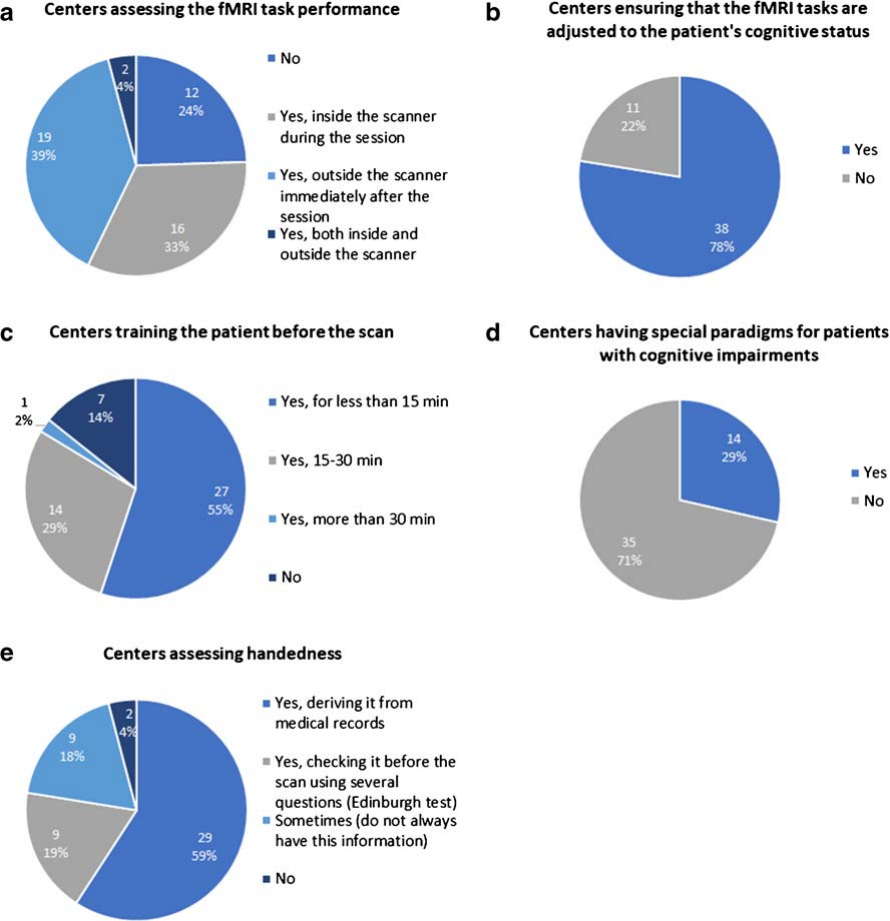
Radiological workflow. **a** Centers assessing the fMRI task performance. **b** Centers ensuring that the fMRI tasks are adjusted to the patient’s cognitive status. **c** Centers training the patient before the scan. **d** Centers having special paradigms for patients with cognitive impairments. **e** Centers assessing handedness

To ensure the accuracy of the exam, most centers assessed the fMRI task performance (37/49, 75.5%) (Fig. 3a), checked that the task was adjusted to the patient’s cognitive status (38/49, 77.6%) (Fig. 3b), trained the patient before scanning (42/49, 85.7%) (Fig. 3c), and assessed the handedness (47/49, 77.6%) (Fig. 3e). However, only a minority of centers had special paradigms for patients with cognitive impairments (14/49, 28.6%) (Fig. 3d).

### Data post-processing protocol and reporting in clinical practice

**Fig. 4:**
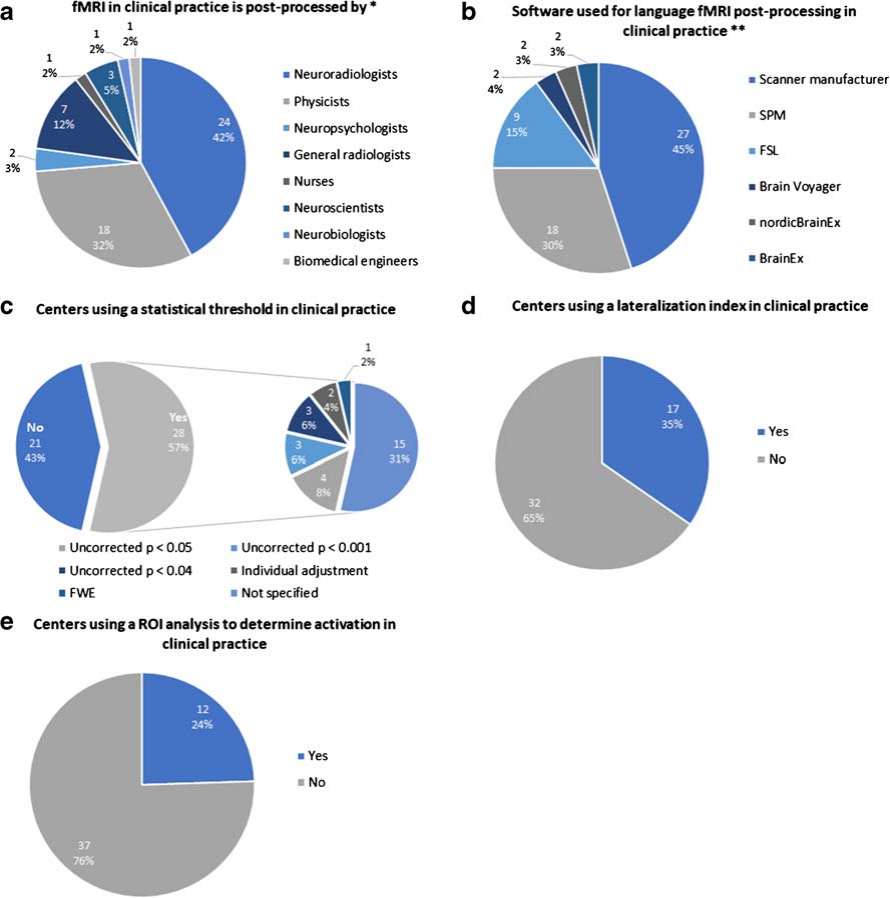
Language fMRI data post-processing. **a** Specialists who post-process fMRI. **b** Software used for post-processing. **c** Centers using a statistical threshold. **d** Centers using a lateralization index. **e** Centers using a ROI analysis to determine activation. ROI region-of-interest, *total responses = 57, **total responses = 60

In most centers, language fMRI was post-processed by radiologists (31/57, 54.4%; 42.1% neuroradiologists and 12.3% general radiologists), or physicists (18/57, 31.6%) (Fig. 4a; note that multiple answers were possible to this question). For ease, we will refer to the whole group as “specialists.”

The main software used for post-processing was CE-certified software provided by the scanner manufacturer (27/60, 45.0%), followed by SPM (Wellcome Department of Imaging Neuroscience, London, UK) (18/60, 30.0%) and FSL (FMRIB Software Library) (9/60, 15.0%) (Fig. 4b; note that multiple answers were possible to this question).

Twenty-eight centers (57.1%) used a fixed statistical threshold (Fig. 4c). Seventeen centers (34.7%) calculated a lateralization index (Fig. 4d) and 12 (24.4%) used a region-of-interest (ROI) analysis (Fig. 4e).

**Fig. 5:**
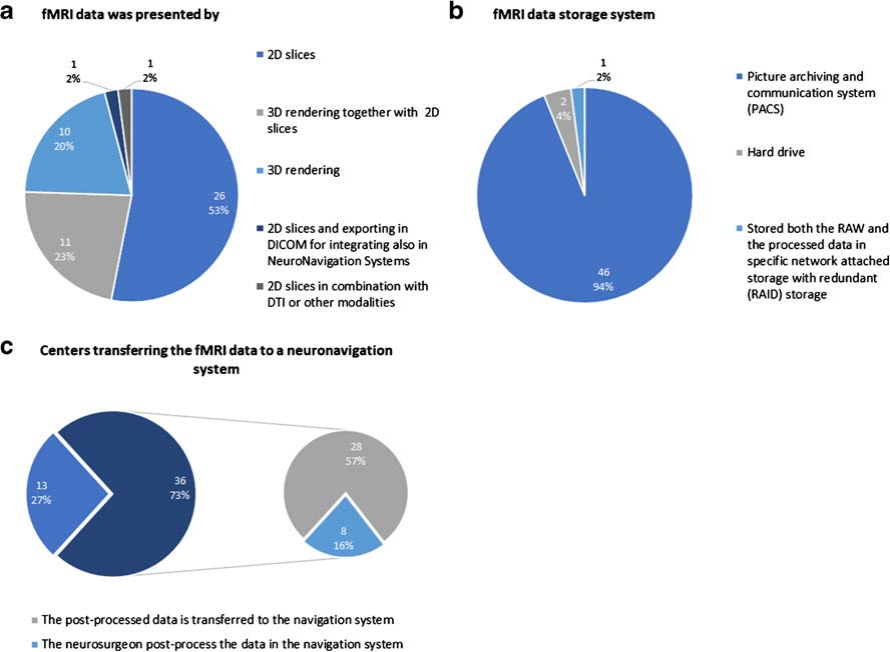
fMRI data presentation, storage and transfer. **a** Presentation format. **b** Storage system. **c** Data transfer to a neuronavigation system

fMRI data was presented using 2D images in most centers (26/49, 53.1%), followed by 3D rendering together with 2D images (11/49, 22.4%) (Fig. 5a). The fMRI data were mainly stored in a picture archiving and communication system (PACS) (46/49, 93.9%) (Fig. 5b). Furthermore, most centers transferred the fMRI data to a neuronavigation system (36/49, 73.4%) (Fig. 5c).

**Fig. 6:**
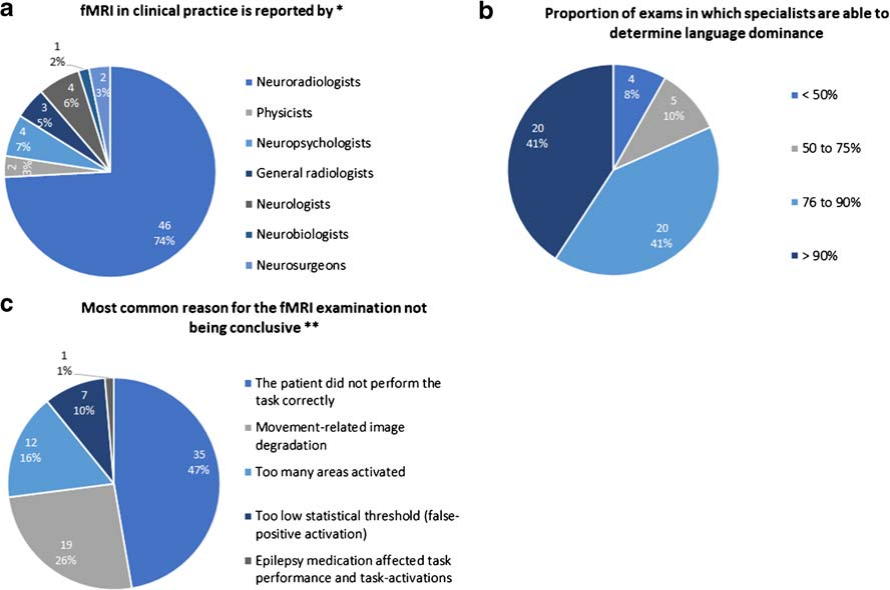
Language fMRI reporting and interpretation. **a** Specialists who report fMRI. **b** Proportion of exams in which specialists are able to determine language dominance. **c** Most common reason for the fMRI examination not being conclusive. *Total responses = 62, **total responses = 74

In most centers, the fMRI examination was reported by a radiologist (49/62, 79.0%; 74.2% neuroradiologists or 4.8% general radiologists) (Fig. 6a; note that multiple answers were possible to this question). The language fMRI interpretation was usually performed by visual inspection (32/49, 65.3%), or by both visual inspection and a lateralization index in 17/49 (34.7%).

Most of the specialists (40/49, 81.6%) were able to determine the language dominance (left, right, or bilateral) in more than 75% of examinations (Fig. 6b). The most common reason for an fMRI examination not to be conclusive was incorrect task performance (35/74, 47.3%), or movement-related image degradation (19/74, 25.7%) (Fig. 6c; note that multiple answers were possible to this question).

Finally, a majority of specialists (38/49) felt confident reporting language fMRI examinations (100% confident: 6/49, 12.2%, and 75% confident; 32/49: 65.3%). Only 8/49 (16.3%) felt 50% confident and 3/49 (6.1%) felt less than 25% confident.

## Discussion

The present survey comprehensively analyzed the current clinical practice of applying fMRI to determine language lateralization in European epilepsy centers. As expected, language fMRI is a routine tool, mainly performed in academic centers, by specialists, mainly neuroradiologists. Although the wide range of paradigms used indicates a low level of standardization, language dominance was determined in a large majority of patients with a high degree of confidence.

This is the first European survey of language fMRI in clinical practice, focusing on epilepsy patients. The survey provides a broad view of the clinical indications of language fMRI as well as the technical aspects of the procedure, its interpretation, and reporting. It was distributed through the ESNR members and the epiCARE imaging group and completed mainly by radiologists (87.8%) highlighting their main role in this imaging technique in Europe.

In the USA, a survey of clinical language fMRI was divided into two sections, one more dedicated to clinical applicability and the other more dedicated to technical aspects. The majority of the respondents were US academic medical centers as well as a few European centers. The clinical section was mostly completed by neurologists and neurosurgeons while the technical section was mainly completed by radiologists (29%), neuropsychologists (25%), and neurologists (25%) [16, 17].

### Generalizability of results

The survey had a reasonable number of responses (49 unique European centers), with a good geographical spread (a total of 20 countries). Most responders were board-certified neuroradiologists working in academic hospitals with an in-house epilepsy surgery service. An overall low participation rate (less than 1% of all invitations) was expected because centers were requested to participate in the survey only if they performed fMRI for language lateralization in epilepsy patients in their clinical practice. This criterion could have favored an overrepresentation of responders with more expertise in language fMRI in epilepsy than in the Wada test, and could have therefore introduced a bias in questions related to the frequency of use of both techniques. The related results should therefore be interpreted with some caution. We consider nevertheless that this survey provides a realistic overview of the clinical practice of the application of language fMRI in people with epilepsy in Europe.

### Clinical practice and indications

The survey confirms that language fMRI is a well-established technique for language lateralization in clinical practice, being the technique of choice among the responding centers, clearly ahead of the Wada test. In fact, most (42.9%) responders of our survey performed between one and five language fMRI scans per month and did not perform any Wada tests. This most probably reflects the inherent advantages language fMRI has over the Wada test, including lower risk, lower cost, and greater potential for localization of function [15]. As stated above, the results of this survey should be interpreted with some caution but they are in line with the results of the previously mentioned survey [16, 17].

The main indication for language fMRI was determining hemispheric dominance and location of language areas in patients with tumors (46.9%) followed by determining hemispheric dominance in patients with epilepsy (26.5%). The latter reflects the recommendation that language fMRI should replace the Wada test in patients with medial temporal lobe epilepsy, temporal lobe epilepsy in general, or extratemporal epilepsy [15].

In Europe, neuroradiologists are the most frequent specialists performing language fMRI, most often supported by MRI physicist; other professionals such as neuropsychologists may be also involved. In contrast, the mainly US covering survey [16] found neuroradiologists and neuropsychologists to be involved in equal proportion. This discrepancy could reflect geographical differences in healthcare provision.

### Language fMRI paradigms and radiological workflow

Thirteen different paradigms for language lateralization were used, highlighting the lack of standardization and pointing to the need for appropriate guidelines. The choice of an optimized paradigm is essential to achieve robust and reproducible results especially in cognitive functions such as language. In general terms, fMRI paradigms are developed by adapting validated neuropsychological tests to the MRI environment, usually using a block design, which however requires the development of a control task. The use of different paradigm protocols can result in different patterns of activation, which could lead to divergent interpretations. Such differences represent a challenge for incorporating language fMRI in the clinical setting and more so when interpreting results from different centers.

Our survey reveals that most centers (87.7%) used more than 1 paradigm to determine language lateralization, most frequently word generation and auditory comprehension tasks. Similarly, Benjamin et al. [16] found that 95% of specialists reported the use of two or more paradigms. This is understandable because language is a complex function which is composed of 5 main domains: listening, speaking, reading, writing and comprehension; it would be therefore challenging to develop a single robust paradigm that activates several language components at the same time. Consideration needs also to be given to the inter- and intra-hemispheric language reorganization that can occur in epileptic patients [10] depending on the location of the pathology. These various confounding factors make it advisable to use at least two paradigms, which activate at least two different language domains. Recently, the American Society of Functional Neuroradiology followed that line by corroborating the recommendation of the use of at least 2 paradigms for presurgical language lateralization [14].

The paradigms most used were word generation tasks (phonemic decision, semantic verbal fluency, and verb generation) followed by comprehensive tasks (auditory and visual comprehensive task and sentence completion). Word generation tasks have been demonstrated to be robust paradigms for assessing language localization and are very effective in activating the frontal gyri of the dominant language hemisphere [14, 18].

They do not, however, regularly activate the temporal cortex [19]. Such activation can be helpful to determining lateralization in those patients in whom an interhemispheric dissociation of frontal and temporal language areas is found. This has been shown to be the case in 3% of 144 epilepsy patients assessed by the Wada test [20, 21]. Auditory comprehension paradigms are best placed to provide superior temporal gyrus activation [14]. Furthermore, they also provide information about semantic and syntactic processes [22, 23].

The semantic decision tasks (sentence completion, verb to noun verb generation, antonymous or synonymous decision task) are the most effective paradigms to activate both the inferior frontal gyrus and superior temporal gyrus [14, 18, 24]. However, some of these paradigms require cognitive skills which are often impaired in the epileptic population [25].

The American Society of Functional Neuroradiology has recommended fMRI paradigm algorithms for surgical language assessment that include word generation tasks, sentence completion, and either object naming or passive story listening in case of an impaired patient. Interestingly enough, they also included a rhyming task as a robust task, which activates Broca and Wernicke areas [14]. This paradigm was not mentioned in this European Survey.

There is a large variation in the control tasks used within each paradigm (see Table 2), which were primarily presented in a visual format. Variations in language fMRI paradigms and instructions could contribute to different patterns of activation between centers, although overall laterality is more likely to remain constant [16].

Most centers (71%) trained the patient before scanning, usually for less than 15 min. This time period could be optimal, as longer stimuli repetition could affect fMRI-based measures of language lateralization [26] and could therefore lead to pseudoincreases in bilateral activation [27]. Most centers also ensured that the fMRI tasks were adjusted to the patients’ cognitive performance, though only a minority used specially adapted paradigms for patients with cognitive impairments. However, having patient-adapted tasks is crucial for obtaining adequate results [28], since excessive complexity of a paradigm could lead to poor activation patterns as a result of underperformance [12].

There was very high convergence on the method used to assess the patient’s performance of the fMRI paradigm, consisting mainly of asking for feedback after the end of the examination, outside the scanner. Compliance with the cognitive tasks is a prerequisite for eliciting the modulation of brain activity on which fMRI depends [15], so checking the fMRI task performance is necessary for the correct interpretation of the examination.

In summary, our survey displays the considerable range of variability of the number and the kind of paradigms used. Even so, the most widely used paradigm is a word generation task (in multiple different versions) and most centers use more than one paradigm to determine language lateralization. In addition, there is an important degree of consistency in the mode of presentation, the training of the patients beforehand, and the adjustment to their cognitive performance.

### Data post-processing protocol and reporting

To explore the degree of standardization of the post-processing pipeline, we queried the software used, the analysis method (qualitative vs quantitative) applied, the way the results were presented, the profession of the person who analyzed them, and the degree of confidence with which the lateralization was determined.

Fifty-five percent of the responding centers used open-source, freely available software such as SPM (Wellcome Department of Imaging Neuroscience, London, UK) or FSL (FMRIB Software Library), while 45.0% used CE-marked image analyses software provided by the scanner manufacturer, which is similar to the results of the US survey (open-source software, 59.0%; manufacturer-provided software scanner, 39.0%) [16]. Among open-source software, SPM was with 30.0% the most frequently used, similar to the 27% of the US survey [16]. Open-source software allows modulation and interrogation of all fMRI post-processing steps, which can lead to time-consuming off-line processing [29]. Open-source software also provides more information than is strictly necessary to determine language lateralization in a clinical context, and is therefore more likely to be used in a research environment. Scanner manufacturer software is usually user friendly and enables real-time observation of the activation, which can be repeated if it is not diagnostic [29]. This software is CE marked and therefore compliant with medical devices regulations which facilitates its use in a clinical environment. The clinical use of non-CE-marked products requires local compliance with a quality control framework to ensure its safe and reproducible application. Therefore, professionals who use SPM or FSL clinically are responsible for the integrity of the product used (e.g., correct designation of right and left). Previous studies comparing MR scanner manufacturer software and SPM found a significant concordance between the two with respect to the area and the intensity of activation confirming that manufacturer software provide adequate and clinically relevant information for patient management [29, 30].

A large number of centers (65%) determined lateralization qualitatively by estimating the difference in hemispheric activation visually. This was also observed in the US survey [17]. Only a minority of centers determined lateralization by both visual inspection and a global lateralization index. Most centers used a predefined statistical threshold, with great variability in the defined threshold value used, in line with the previously mentioned survey [16]. Lateralization indices and ROI analyses were only used by a limited number of centers. Predefined ROIs are used to determine activation in defined regions; they can guide evaluation of the target parameter and exclude unspecific activation from lateralization indices [31, 32]. However, ROIs are often defined on the basis of examinations in healthy people, which can be problematic when language areas are displaced from their typical location in epilepsy patients [33].

fMRI examinations were most frequently post-processed and reported by neuroradiologists, while other professionals (neuropsychologists, neurologists, general radiologists, neurosurgeons, physicists, neurobiologists, neuroscientists, biomedical engineers, and nurses) were less frequently involved. Data post-processing and reporting require a set of skills which include knowledge of neuroanatomy, structural bases of cognition, MR physics, image artifacts, statistical analysis, and the use and development of psychological tests [16, 34]. The training in these skills is a necessary prerequisite for a correct interpretation of the results.

To support this interpretation, handedness was assessed in 77.6%, either by medical records (59.2%) or questionnaires (18.4%). Handedness has been proposed as a possible mediator of an atypical pattern of language lateralization (bilateral or right-hemispheric language lateralization) in patients with epilepsy [22, 35, 36]; recording handedness would be therefore very useful to the correct interpretation of studies.

Finally, most specialists (75%) felt confident reporting language fMRI, in line with previous results [17]. Inability to determine lateralization (0–25%) was mostly attributed to the fact that the patient did not perform the task correctly, emphasizing the need for using patient-adapted tasks [12, 28].

In summary, our survey shows that there is a considerable reliance on non-CE-marked post-processing analysis methods, mainly post-processed by neuroradiologists, and most of the examinations are analyzed qualitatively through visual inspection. Lateralization is mostly determined with a high degree of confidence.

## Conclusion

This first survey of language lateralization fMRI of European centers shows that language fMRI is a clearly established clinical tool used to determine language lateralization in the preoperative management of epilepsy patients. Analysis, reporting, and correctly and confidently identifying the lateralized hemisphere are mostly performed by neuroradiologists. Despite this clear clinical activity, there is, however, a large degree of variability in the paradigms used as well as the post-processing software applied, which was commonly not CE marked. The latter most probably reflects the fact that most of these examinations are performed in academic health centers, with a large experience in research as well as in clinical management of epilepsy patients. Changing legislation will make it more difficult in the future to use non-CE-marked products in patient care. Thus, the need to ensure the best possible outcome for the individual patient and the need to improve transparence and comparability of clinical outcome data of each center will require the setting of some standards which the community will follow and which the industry can create the appropriate software tools for.

## Electronic supplementary material

ESM 1 (DOCX 86 kb)
