# Supplementary material for: Clinical practice of language fMRI in epilepsy centers: a European survey and conclusions by the ESNR Epilepsy Working Group
Source: Neuroradiology. 2020 Mar 13;62(5):549–62. doi: 10.1007/s00234-020-02397-w (PMC7186249; doi:10.1007/s00234-020-02397-w)
Supplement: Supplementary file 1 — (DOCX 86 kb) [file 234_2020_2397_MOESM1_ESM.docx]

**ESNR Epilepsy Working Group Member Survey 2017**

This is a survey prepared by the Epilepsy Working group of the Diagnostic Committee of the ESNR. The goal of this survey is to collect information about how the fMRI for language lateralization in epilepsy patients is performed around Europe.

We kindly ask you to answer all the question that you are able to so that we can better understand the workflow of fMRI for language lateralization in epilepsy patients at your institution.

*Required

## Email address *

1. **Variable assessed: Demographic information**

Please note that some of the questions can have multiple answers.

## 1.1.) Question: Which best describes you? *

*Tick all that apply.*

Neuroradiologist in training (resident/fellow) Board certified general radiologist

Board certified neuroradiologist

Other

## If answering other, please specify:

**1.2) Question: Which best describes your current employment? ***

*Mark only one oval.*

General Hospital Academic hospital Other

## If answering other, please specify:

**1.3) Question: Which country are you currently employed in? ***

*Mark only one oval.*

Afghanistan Akrotiri Albania Algeria

American Samoa Andorra

Angola Anguilla Antarctica

Antigua and Barbuda Argentina

Armenia

Aruba

Ashmore and Cartier Islands Australia

Austria

Azerbaijan Bahamas, The Bahrain Bangladesh Barbados Bassas da India Belarus Belgium

Belize Benin Bermuda Bhutan Bolivia

Bosnia and Herzegovina Botswana

Bouvet Island

Brazil

British Indian Ocean Territory British Virgin Islands

Brunei

Bulgaria Burkina Faso Burma Burundi Cambodia

Cameroon Canada Cape Verde

Cayman Islands

Central African Republic Chad

Chile

China

Christmas Island Clipperton Island Cocos (Keeling) Islands Colombia

Comoros

Congo, Democratic Republic of the Congo, Republic of the

Cook Islands Coral Sea Islands Costa Rica

Cote d'Ivoire Croatia Cuba Cyprus

Czech Republic Denmark Dhekelia Djibouti Dominica

Dominican Republic Ecuador

Egypt

El Salvador Equatorial Guinea Eritrea

Estonia Ethiopia Europa Island

Falkland Islands (Islas Malvinas) Faroe Islands

Fiji

Finland France

French Guiana

French Polynesia

French Southern and Antarctic Lands Gabon

Gambia, The Gaza Strip Georgia Germany Ghana Gibraltar

Glorioso Islands Greece Greenland Grenada Guadeloupe Guam Guatemala Guernsey Guinea

Guinea-Bissau Guyana

Haiti

Heard Island and McDonald Islands Holy See (Vatican City)

Honduras Hong Kong Hungary Iceland India Indonesia Iran

Iraq Ireland Isle of Man Israel

Italy Jamaica Jan Mayen Japan Jersey Jordan

Juan de Nova Island

Kazakhstan

Kenya Kiribati Korea, North Korea, South Kuwait Kyrgyzstan Laos

Latvia Lebanon Lesotho Liberia Libya

Liechtenstein Lithuania Luxembourg Macau Macedonia Madagascar Malawi Malaysia Maldives Mali

Malta

Marshall Islands Martinique Mauritania Mauritius Mayotte

Mexico

Micronesia, Federated States of Moldova

Monaco Mongolia Montenegro Montserrat Morocco Mozambique Namibia Nauru

Navassa Island Nepal

Netherlands

Netherlands Antilles New Caledonia New Zealand Nicaragua

Niger Nigeria Niue

Norfolk Island

Northern Mariana Islands Norway

Oman

Pakistan Palau Panama

Papua New Guinea Paracel Islands Paraguay

Peru Philippines Pitcairn Islands Poland Portugal Puerto Rico Qatar

Reunion Romania Russia Rwanda Saint Helena

Saint Kitts and Nevis Saint Lucia

Saint Pierre and Miquelon

Saint Vincent and the Grenadines Samoa

San Marino

Sao Tome and Principe Saudi Arabia

Senegal Serbia Seychelles Sierra Leone

Singapore

Slovakia Slovenia Solomon Islands Somalia

South Africa

South Georgia and the South Sandwich Islands Spain

Spratly Islands Sri Lanka Sudan Suriname Svalbard Swaziland Sweden Switzerland Syria

Taiwan Tajikistan Tanzania Thailand Timor-Leste Togo Tokelau Tonga

Trinidad and Tobago

Tromelin Island Tunisia

Turkey Turkmenistan

Turks and Caicos Islands Tuvalu

Uganda Ukraine

United Arab Emirates United Kingdom United States Uruguay

Uzbekistan Vanuatu Venezuela Vietnam

Virgin Islands

Wake Island Wallis and Futuna West Bank Western Sahara Yemen

Zambia Zimbabwe

## 1.4) Question: Is epilepsy surgery performed at your institution? *

*Mark only one oval.*

Yes No

## 1.5) Question: Please provide the name of your institution to avoid duplicate information from the same source. This will be kept entirely confidential and will not be included in the survey results or reporting. Alternatively, please certify that you are providing information as the single representative of your institution. *

**1.6) Question: Are your performing fMRI for language lateralization in epilepsy patients in clinical practice (not research)? ***

*Tick all that apply.*

yes No

# If you have answered no, please do not fill the survey. We thank you for collaboration.

1. **Variable assessed: Institution workflow**

Please note that some of the questions can have multiple answers.

## 2.1) Question: At your institution and in clinical practice, how is the language lateralization studied? *

*Tick all that apply.*

Language fMRI Wada Test Other

## If answering other, please specify:

**2.2) Question: At your institution, approximately how many fMRI exams in clinical practice are done per month ? ***

*Mark only one oval.*

Not sure

< 1

1-4

> 5

## 2.3) Question: At your institution, approximately how many fMRI exams in clinical practice are done in epilepsy patients per month? *

*Mark only one oval.*

Not sure

< 5

5-10

> 10

## 2.4) Question: At your institution, what is the main indication for language fMRI in clinical practice? *

*Tick all that apply.*

Not sure

Language lateralization in epilepsy patients

Language lateralization and location of language areas in patients with a tumor

## 2.5) Question: If you perform Wada test, how many are performed in your institution per year?

*Mark only one oval.*

Not sure

Less than 5 per year Between 5-15 per year More than 15 per year

## 2.6) Question: Which of the following describes your institution? *

*Mark only one oval.*

No MRI physicist support

General MRI physicist support (e.g. 1 physicist supporting body and neuroimaging) Specialist neuro MRI physicist support

# Variable assessed: Radiological workflow

Please note that some of the questions can have multiple answers.

## 3.1) Question: Which specialist performs the fMRI in clinical practice? *

*Tick all that apply.*

Neuroradiologist Physicist Psychologists

More than two specialists

## If you answered "more than two specialists", please provide more information:

**3.2) Question: In clinical practice, do you ensure that the fMRI tasks are adjusted to the patient's cognitive status, for instance by referring to the patient’s medical records? ***

*Mark only one oval.*

Yes No

## 3.3) Question: In clinical practice, do you allow the patient to train the tasks before the scan? *

*Mark only one oval.*

Yes No

## 3.4) Question: If you have answered yes, how long do you spend training the patient?

*Mark only one oval.*

Less than 15 minutes

Between 15 minutes and 30 minutes More than 30 minutes

## 3.5) Question: In clinical practice, do you check the handedness dominance of the patient? *

*Mark only one oval.*

Yes. We always look for it in the medical records

Yes, we check before the scan using several questions (Edinburgh test) Sometimes, we do not always have this information

No

## 3.6.) Question: In clinical practice, do you have adapted paradigms for patients with cognitive impairment? *

*Mark only one oval.*

Yes No

# Variable assessed: fMRI experiment

Please note, that some of the questions can have multiple answers.

## 4.1) Question: In clinical practice, how many paradigms do you use for language lateralization? *

*Mark only one oval.*

1

2

3

4 or more

## 4.2) Question: In clinical practice, do you use a specific paradigm for specific activation of the inferior frontal area and superior temporal area separately or do you use a paradigm that activates both areas simultaneously? *

*Mark only one oval.*

Separate paradigm for each region (temporal and frontal) Paradigm which provide activation of both regions

# Please, describe the type of paradigm (Verbal fluency, Word fluency, Verbal Comprehension, etc):

## For inferior frontal area:

**For superior temporal area:**

**For both areas simultaneously:**

**4.3) Question: In clinical practice, do you use other paradigms to activate other areas than frontal inferior or temporal superior? ***

*Mark only one oval.*

Yes No

# If answering yes, please specify:

## Which area?

**Which paradigm?**

**4.4) Question: How are the stimuli presented? ***

*Tick all that apply.*

Visual Auditory Other

## If answering other, please specify:

**4.5) Question: If the patient is collaborative and has to generate words, how does he/she perform the task? ***

*Tick all that apply.*

In silence, without pronouncing the words Speaking normally

Whispering

The patient does not generate words in any of the paradigms used

## 4.6) Question: In clinical practice, do you control the fMRI task performance? *

*Mark only one oval.*

Yes No

# If you have answer yes, please answer the two following questions.

## 4.7) Question: Where do you control the task performance?

*Mark only one oval.*

Inside the scanner during the session

Outside the scanner, immediately after the session Other

## If answering other, please specify:

**4.8) Question: In clinical practice, how do you monitor the performance ?**

*Mark only one oval.*

By button response during the session

Listening to the words with a microphone during the session

Asking the patient outside the scanner after the performance of the task I do not monitor the performance

Other

Other:

## If answering other, please specify:

**Please, make a short description of the most common paradigms used, describing the activation task and the baseline task**

**Paradigm 1**

**Activation Task**

**Baseline Task**

**Paradigm 2**

**Activation Task**

**Baseline Task**

**Paradigm 3**

**Activation Task**

**Baseline Task**

1. **Variables assessed: Post-processing and reporting**

Please note that some of the questions can have multiple answers.

## 5.1) Question: Who does the post-processing in clinical practice? *

*Tick all that apply.*

Physicist Neuroradiologist Radiographer Other

## If answering other, please specify:

**5.2) Question: In clinical practice, which software are you using for the post-processing ?**

*****

*Tick all that apply.*

SPM

Brain Voyager FSL

Scan Manufacter Other

## If answering other, please specify:

**5.3) Question: In clinical practice, do you use a statistical threshold below which you reject the examination or state in the report the problem of false positive activation? ***

*Mark only one oval.*

Yes No

## If answering yes, please state details:

**5.4) Question: In clinical practice, do you use a lateralization index (counting the number of voxels)? ***

*Mark only one oval.*

Yes No

## 5.5) Question: In clinical practice, do you use a Region of Interest (ROI) analysis to determine the activation in defined regions? *

*Mark only one oval.*

Yes No

## If answering yes, please state which regions are included in the ROI:

**5.6) Question: Who interprets/reports the fMRI examination in clinical practice? ***

*Tick all that apply.*

Neuroradiologist Neurologist Neuropsychologist Other

## If answering other, please specify:

**5.7) Question: In clinical practice, how do you interpret the fMRI examination? ***

*Tick all that apply.*

Visual inspection

Global index lateralization including all the hemispheres and cerebellum Index lateralization using a ROI of the temporal and frontal lobes

Both, visual inspection and lateralization index

## 5.8) Question: In clinical practice, how do you present the fMRI data? *

*Tick all that apply.*

3D rendering 2D slices Other

## If answering other, please specify:

**5.9) Question: In clinical practice, how do you store the fMRI data? ***

*Mark only one oval.*

PACS

Other

## If answering other, please specify:

**5.10) Question: Do you transfer the fMRI data to the neuronavigation system? ***

*Mark only one oval.*

Yes No

## 5.11): Question: If you have answered yes, please explain how do you send the fMRI data to the neuronavigation system?

*Mark only one oval.*

I send the fMRI data post-processed

The neurosurgeon post-process the data in the navigation system

## 5.12) Question: In what proportion of fMRI examinations are you unable to determine the language dominance? *

*Mark only one oval.*

In less than 10% of cases About 10 to 25% of cases About 25-50% of cases In more of 50% of cases

## 5.13) Question: In your opinion, which is the most common reason for the fMRI examination not being conclusive ? *

*Tick all that apply.*

The patient does not perform the task correctly There are too many areas activated

The statistical threshold is too low and false positive activation areas are generated

The images are degraded by movement artifacts Other

## If answering other, please specify:

**5.14) How confident do you feel when you report an fMRI exam for language lateralization in epilepsy patient? ***

*Mark only one oval.*

100% confident

75% confident

50% confident

less 25% confident

# THANK YOU FOR YOUR COLLABORATION. THE RESULTS OF THE SURVEY WILL BE PRESENTED AT AN UPCOMING ESNR MEETING. THE ESNR EPILEPSY WORKING GROUP WILL USE THIS DATA TO DEVELOP RECOMMENDATIONS ON PERFORMING FMRI LANGUAGE LATERALIZATION EXAMINATIONS IN EPILEPSY PATIENTS.
